# Diagnostic and Prognostic Value of Thrombocytopenia in Severe Burn Injuries

**DOI:** 10.3390/diagnostics14060582

**Published:** 2024-03-09

**Authors:** Eliza-Maria Bordeanu-Diaconescu, Andreea Grosu-Bularda, Adrian Frunza, Sabina Grama, Mihaela-Cristina Andrei, Tiberiu-Paul Neagu, Ioan Lascar, Cristian-Sorin Hariga

**Affiliations:** 1 Department of Plastic Surgery and Reconstructive Microsurgery, “Carol Davila” University of Medicine and Pharmacy Bucharest, 010825 Bucharest, Romania; eliza.diaconescu@umfcd.ro (E.-M.B.-D.); cristian.hariga@umfcd.ro (C.-S.H.); 2 Burn Centre, Emergency Clinical Hospital of Bucharest, 014461 Bucharest, Romania

**Keywords:** severe burns, platelets, thrombocytopenia, sepsis, mortality

## Abstract

Background and objectives: Burn injuries are the most severe type of trauma, with complex biological consequences associated with high rates of morbidity and mortality. Prompt recognition and management of burn-related complications are imperative for improving the vital and functional prognosis of the patient. Changes in biological parameters can be essential determinants in the prognosis of the burned patient. Thrombocytopenia in critically ill patients is linked to an elevated risk of mortality. We sought to investigate the significance of thrombocytopenia in severely burned patients while considering the limited available data in the literature. Materials and methods: A two-year retrospective study was conducted on 90 patients with severe burns admitted to our Burn Centre. Demographic data, burn lesion characteristics, and daily total blood counts, including platelet assessment, complications, and mortality, were recorded and analyzed. Results: Patients with extensive burns in our study had a poor prognosis based on their Abbreviated Burn Severity Index score (ABSI), age, percentage of total body surface area (TBSA) burned, presence of third-degree burns, and inhalation injuries. Regardless of the moment, patients with thrombocytopenia in our study died significantly more frequently. Compared with the survivors, the platelet count was significantly lower at any given time in the non-survivors group. Significant statistical associations between thrombocytopenia and ABSI score, burn surface area, presence of third-degree burns, and inhalation injuries were identified at different timeframes post-burn injury. Sepsis was encountered in one-third of the patients. Thrombocytopenia was more frequent in patients with sepsis who did not survive compared to survivors and did not normalize until the time of death. Conclusions: Thrombocytopenia represents an early indicator of severe complications and outcome predictor in severely burned patients. It is correlated with recognized negative prognostic factors and also with sepsis occurrence. Future research efforts should focus on refining early detection parameters and interventions to improve the prognosis of burn patients.

## 1. Introduction

Burns are the most severe type of trauma, with complex biological consequences associated with high rates of morbidity and mortality, even in specialized burn centers. Critical care management and local treatment are essential in the case of severe burns, requiring assistance provided by a multidisciplinary team. Prompt identification of complications is essential, determining the accurate treatment and enhancing both the patient’s vital and functional prognosis. Changes in biological parameters play a crucial role in the prognosis of burn patients [1,2].

Platelets, also known as thrombocytes, are small, disc-shaped blood cells that play a crucial role in blood clotting and wound healing. They are produced in the bone marrow from large, multinucleated precursor cells called megakaryocytes and have an average lifespan in the bloodstream ranging from 7 to 14 days. Platelets contain different types of granules that contain a variety of bioactive molecules, such as clotting factors, growth factors, and inflammatory mediators [3,4,5].

Although studies have demonstrated that thrombocytopenia in critically ill patients is associated with increased mortality risk, there is limited literature as to the significance of thrombocytopenia in severely burned patients [6,7]. Thrombocytopenia can be seen in burn patients due to several factors: hemodilution in the initial phases of burn injury associated with massive fluid resuscitation, activation and consumption of platelets due to the extensive tissue damage and inflammation, bone marrow suppression, medications such as antibiotics, and disseminated intravascular coagulation. Infections lead to a wasteful process involving platelets and are one of the main causes of thrombocytopenia in burn patients [8].

Several studies showed a significant and progressive decrease in total platelet count (TPC) in non-surviving patients with massive burns. On the contrary, in the surviving patients, after the initial decline, there is a rebound rise in platelet counts, and most patients have a normal platelet count before their discharge [3,9,10].

Extensive clinical investigations carried out in intensive care units indicate that thrombocytopenia serves as a predictor for both mortality and the development of multiple organ failure in sepsis cases [11,12]. Thrombocytopenia occurs in approximately 35–40% of patients within intensive care units, with sepsis being the primary contributor, accounting for an incidence rate exceeding 50% in critical patients [13,14].

Burns have traditionally been excluded from significant investigations into sepsis diagnosis and treatment because the hypermetabolic state and systemic inflammation present in burn patients interfere with identifying the typical clinical signs of sepsis [15]. Despite the critical significance of infections and sepsis, diagnosing and predicting them continues to pose a significant challenge. Currently, no definitive diagnostic criteria or reliable predictive formulas are available to foresee the onset of sepsis and infections accurately [16]. The Surviving Sepsis After Burns Campaign 2023 consensus strongly recommends against using Systemic Inflammatory Response Syndrome (SIRS) criteria as an early warning for sepsis and considers an acute drop in platelet count to be a trigger for considering the diagnosis of sepsis [17]. SIRS is a clinical syndrome characterized by a widespread inflammatory response throughout the body and can be triggered by various insults such as infection, trauma, ischemia, and other inflammatory conditions. The criteria for SIRS were established by the American College of Chest Physicians and the Society of Critical Care Medicine in 1992. To diagnose SIRS, a patient must meet at least two of the following criteria: either fever (>38°) or hypothermia (temperature < 36°), tachycardia (heart rate > 90 beats per minute), tachypnea (respiratory rate > 20 breaths per minute), and either leukocytosis (white blood cell count > 12,000/microliter) or leukopenia (<4000/microliter) or the presence of more than 10% immature forms of white blood cells [18].

The objective of our study was to examine the progression of platelet count fluctuations in individuals with burn injuries and to assess the relationship between the development of thrombocytopenia and various patient and burn-related variables. To achieve this, we gathered and evaluated data on patient characteristics, the extent of burn injuries, and mortality rates.

## 2. Materials and Methods

A two-year retrospective study was conducted on 90 patients with severe burns admitted to the Burn Centre of the Clinical Emergency Hospital in Bucharest, Romania, from January 2018 to December 2019. The inclusion criteria for patients were as follows: age ≥ 18 years old, partial-thickness burns greater than 25% or full-thickness burns greater than 20% percent of the total body surface area, and admission to our hospital within the first 48 h after the burn injury. Exclusion criteria were: partial-thickness burns <25% TBSA or full-thickness burns <20% TBSA, late admission to our Burn Centre (>48 h), a transfer to another hospital, patients with known pre-existing hematological disease, and cases with incomplete clinical data and/or laboratory tests.

The following demographic and clinical characteristics of the included patients were obtained: thrombocyte count, age, gender, TBSA, third-degree burn, inhalation injuries, results of microbiological assessment, and mortality within 60 days post-burn injury. Abbreviated Burn Severity Index (ABSI) score was also calculated [19].

Thrombocytopenia was defined as a platelet count below 150,000/microliter and thrombocytosis above 450,000/microliter of blood [20].

For extensive burns (>20% TBSA), intravenous fluid resuscitation was commenced upon admission, following the Parkland formula, which is used to determine the optimal fluid volume necessary for rehydration and prevention of insufficient intravascular fluid causing hypoperfusion, decreased cardiac output, and consequently cardiogenic shock and tissue damage causing additional harm to burn victims. The formula dictates the administration of 4 mL of Lactated Ringer’s solution per patient’s weight in kilograms, multiplied by the percentage of total body surface area affected by the burn injury. This approach served as a guideline for determining the appropriate fluid volume needed during the initial 24 h after a burn incident, with half of the calculated volume administered within the first eight hours after the burn. The fluid regimen was promptly adjusted to ensure adequate perfusion endpoints, such as urine output of over 0.5 mL/kg/h, preventing both under-resuscitation and over-resuscitation [21]. Bronchoscopy was used for early evaluation of upper airway injury.

The treatment of burns in our Burn Centre involved a multidisciplinary approach and aimed to address the various aspects of burn injury, including continuous monitoring of vital signs, fluid balance, and electrolytes; wound care; pain management; infection control; nutritional support; and support of organ function as needed, especially in cases where burns affected multiple organ systems.

Daily blood tests, including complete blood count, were ordered to assess a patient’s status, track disease progression, or monitor the effects of treatments.

From the time of admission, escharotomies were undertaken in patients presenting with circumferential burns with limb compression and circumferential burns of the thoracic wall, resulting in respiratory compromise by restricting normal chest wall movement. For patients with full-thickness burns, the approach involved excision and skin grafting using autografts. We conducted daily care for burn wounds, and the dressing procedures followed the standardized protocols established within our unit.

Microbiological screening was performed at admission, and repeated cultures were obtained to monitor changes in the microbial flora over time. In case of suspected infection, microbiological assessment consisted of burn wound cultures, tracheal aspirate cultures, blood cultures (both aerobic and anaerobic), and urinary cultures, depending on clinical findings and paraclinical investigations. Cases of burn wound infections, pneumonia, bloodstream infections, and urinary tract infections were recorded, and the criteria outlined by the American Burn Association for sepsis were employed to identify patients with sepsis [22]. In cases of documented infections, antimicrobial therapy was guided by antibiograms accordingly.

Except in cases with contraindications to anticoagulation, all patients received pharmacological thromboprophylaxis with 40 mg of Enoxaparin subcutaneously once a day. In cases of severe thrombocytopenia, <50,000/μL of thromboprophylaxis was withheld and restarted at the critical care doctor’s indication.

Statistical analysis was performed using IBM SPSS Statistics 25 and Microsoft Office Excel/Word 2013. The distribution of data was tested using the Shapiro–Wilk test. Quantitative variables were expressed as means ± standard deviation (SD) and medians ± standard deviation (SD). Mann–Whitney U-Test was used with nonparametric data, whereas the Student’s *t*-test was used with data that met the assumptions associated with parametric distributions. Cox proportional hazard regressions were used to estimate the hazard ratio (HR) for the occurrence of death, and 95% confidence intervals were calculated. Differences in frequencies of qualitative variables in the contingency tables were tested using the Pearson Chi-Square or Fisher’s Exact Test alongside performing Z tests with Bonferroni correction to detail the results. *p* values of <0.05 were considered statistically significant.

## 3. Results

**Figure 1:**
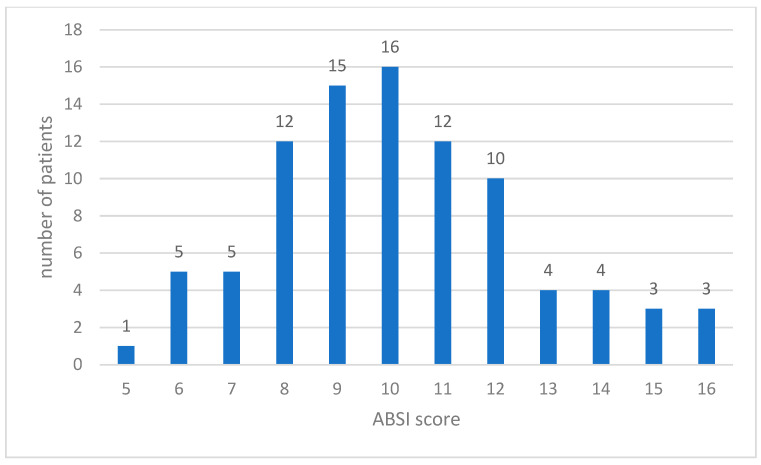
Distribution of patients with regard to the ABSI score.

A total of 90 patients were included in the current study. In the 60-day follow-up time post-burn, 58 patients (64.4%) died (2 of them in the first 48 h), and 32 patients (35.6%) survived. The mean overall age of the patient cohort was 57.2 ± 18.3 (range of 20–90 years). The majority of patients were in the 61–80 years age group (34.4%), followed by patients in the 41–60 years age group (27.8%). The mean percentage of total body surface area (TBSA) burned was 48.7 ± 20.1% (range 20–98%) (Table 1). The mean ABSI score was 10.16 ± 2.47 (range 5–16), with 28 patients having an ABSI score between 10 and 11 (meaning a probability of survival of 20–40%) and 24 patients having an ABSI ≥ 12 (probability of survival ≤ 10%) (Figure 1).

**Figure 2:**
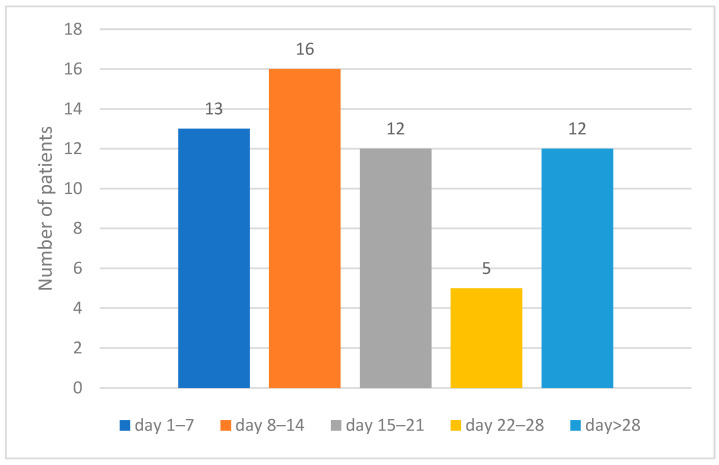
Time of death in patients included in the study.

Among the 58 deaths, 13 deaths appeared in the first week post-burn injury (2 deaths in the first 48 h), 16 deaths in the second week post-burn injury, 12 deaths in the third week, 5 deaths in the fourth week, and 12 deaths after the first 4 weeks post-burn injury (Figure 2).

The mean number of days post-burn injury until death was 22.41 ± 23.89 days, with a median of 14 days (IQR 7.75–26.25).

**Figure 3:**
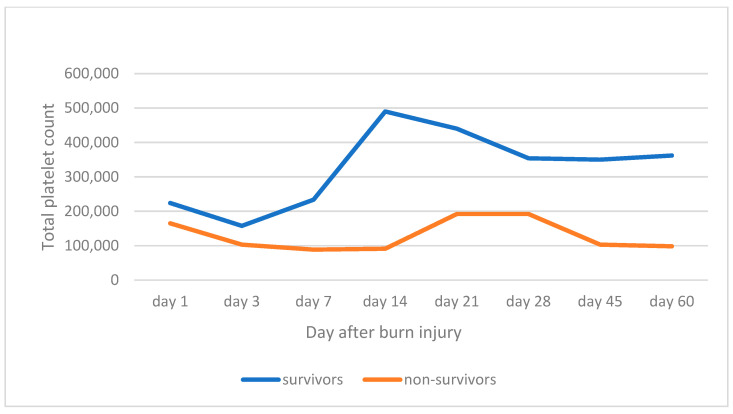
Evolution of platelet counts over time in survivors and non-survivors.

In both survivors and non-survivors, the platelet count was lower on day 3 than at admission, with a median platelet count of 157,500/microliters in survivors and 103,000/microliters in non-survivors. In survivors, the platelet count started to rise after day 3 post-burn injury. In non-survivors, the median platelet count persisted in being low, with a platelet count of 88,500/microliters on day 7 and 91,000/microliters on day 14, and started to increase after day 14 (Figure 3).

The data in Table 2 represent the distribution of patients related to the presence of thrombocytopenia on days 3, 7, 14, 28, 45, and 60 and survival. Survivors discharged within the 60-day timeframe exhibited normal platelet counts at the time of discharge, and any subsequent data regarding their platelet counts were omitted from the study analysis. The differences observed between the groups are significant according to the Fischer test, noting that regardless of the moment, patients with thrombocytopenia died significantly more frequently (*p* < 0.05).

Thrombocytopenia on day 3 post-burn injury was associated with shorter survival periods (39.4 days, 95% CI: 28.9–49.9) compared to patients with normal platelet counts (65.1 days, 95% CI: 46.3–83.9) according to the Log-rank test (*p* = 0.025). In the Cox regression analysis, thrombocytopenia on day 3 postburn was predictive of mortality, with patients with thrombocytopenia having a 2.081 times higher mortality risk (95% CI: 1.07–4.03) (*p* = 0.030) (Table 3).

Thrombocytopenia on day 7 post-burn injury was associated with shorter survival periods (30.1 days, 95% CI: 20.8–39.3) compared to patients with normal platelet counts (80.1 days, 95% CI: 64–96.2) according to the Log-rank test (*p* < 0.001). In the Cox regression analysis, thrombocytopenia on day 7 postburn was predictive for mortality, with patients with thrombocytopenia having a 5.89 times higher mortality risk (95% CI: 2.7–12.6) (*p* < 0.001) (Table 3).

Thrombocytopenia on day 14 post-burn injury was associated with shorter survival periods (27.1 days, 95% CI 17.2–37.1) compared to patients with normal platelet counts (75.9 days, 95% CI: 62.9–88.9) according to the Log-rank test (*p* < 0.001). In the Cox regression analysis, thrombocytopenia on day 14 postburn was predictive of mortality, with patients with thrombocytopenia having a 6.16 times higher mortality risk (95% CI: 3–12.6) (*p* < 0.001) (Table 3).

Thrombocytopenia on day 21 post-burn injury was associated with shorter survival periods (34.3 days, 95% CI 24.4–44.2) compared to patients with normal platelet counts (83.9 days, 95% CI: 69.9–97.8) according to the Log-rank test (*p* < 0.001). In the Cox regression analysis, thrombocytopenia on day 21 postburn was predictive of mortality, with patients with thrombocytopenia having a 9.61 times higher mortality risk (95% CI: 3.3–27.5) (*p* < 0.001) (Table 3).

Thrombocytopenia on day 28 post-burn injury was associated with shorter survival periods (42.2 days, 95% CI: 30.8–53.7) compared to patients with normal platelet counts (84.4 days, 95% CI: 69.8–99) according to the Log-rank test (*p* < 0.001). In the Cox regression analysis, thrombocytopenia on day 21 postburn was predictive of mortality, with patients with thrombocytopenia having a 6.63 times higher mortality risk (95% CI: 1.9–22) (*p* = 0.002) (Table 3).

Data in Table 4 show that patients with higher ABSI scores had thrombocytopenia on days 3, 7, and 14 (*p* < 0.001). There was no obvious difference in platelet levels in the following periods in relationship with the ABSI score.

Older patients had significantly lower platelet counts at any point in time, except on day 21, with a strong association in the first 7 days post-burn (*p* = 0.002) (Table 5)

On day 3, the total burn surface area distribution was non-parametric in both groups according to the Shapiro–Wilk test (*p* < 0.05). The differences between groups were significant according to the Mann–Whitney U test (*p* = 0.004), so patients with higher total body surface area burned (median = 50%, IQR = 35–70%) had thrombocytopenia on day 3 after the burn injury more often than patients with a smaller TBSA burned (median = 35%, IQR = 27.5–40%). On day 7, the total burn surface area distribution was non-parametric in both groups according to the Shapiro–Wilk test (*p* < 0.05). The differences between groups were significant according to the Mann–Whitney U test (*p* = 0.001), so patients with a higher total body surface area burned (median = 50%, IQR = 35–70%) had thrombocytopenia on day 7 after the burn injury more often than patients with a smaller TBSA burned (median = 35%, IQR = 25–42.5). Data analysis on day 14 and afterward showed no statistical significance comparing the TBSA and platelet count (Table 6).

Data analysis showed that patients with thrombocytopenia on day 3 and day 7 post-burn injury had significantly more frequent third-degree burns (74.2% vs. 45.5%, respectively; 67.7% vs. 36.4%) (*p* = 0.019 vs. *p* = 0.013, respectively). Thrombocytopenia on day 14 and afterward was not significantly more frequently associated with third-degree burns (*p* > 0.05) (Table 7).

Data analysis showed that patients with third-degree burns had thrombocytopenia on day 3 and day 7 post-burn injury significantly more frequently than patients without third-degree burns (74.2% of patients with third-degree burns vs. 45.5% of patients without third-degree burns; 67.7% vs. 36.4%, respectively) (*p* = 0.019 and *p* = 0.013, respectively). Third-degree burns were not significantly more frequently associated with thrombocytopenia on day 14 post-burn injury and afterward (*p* > 0.05) (Table 7).

Patients with inhalation injury had thrombocytopenia on day 7 post-burn more frequently than patients without inhalation injury (67.8% vs. 40%) (*p* = 0.028) (Table 7).

**Figure 4:**
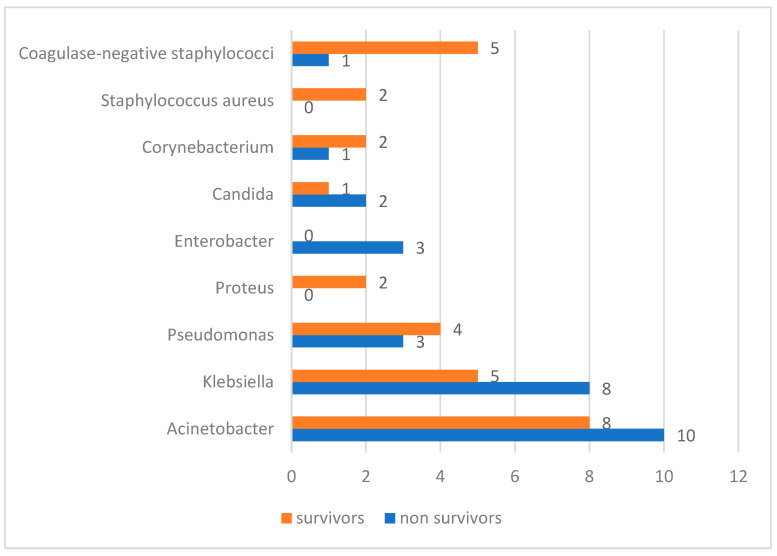
Microorganisms causing sepsis in survivors and non-survivors.

Of the 90 patients, 30 patients had positive blood cultures for at least one period: 18 of the deceased patients and 12 of the survivors. Based on the positive hemocultures and clinical picture, a diagnosis of sepsis was made. In the case of deceased patients, 10 of the 18 patients with positive hemocultures (55.5%) had thrombocytopenia at the time of sepsis diagnosis, and platelet levels did not improve until death in these patients. In four patients who had normal platelet levels at the time of diagnosis of sepsis, thrombocytopenia occurred within 7 days. In surviving patients, 2 of the 12 patients with positive hemocultures (16.6%) had thrombocytopenia at the time of sepsis diagnosis, which normalized within the following days. We also examined the microorganisms responsible for the sepsis, the distribution being presented in Figure 4. A series of ESKAPE pathogens were encountered predominantly in the non-survivor group of patients.

## 4. Discussion

Severe burns trigger a complex cascade of events, including immune and inflammatory responses, metabolic alterations, and burn-induced coagulopathy. Despite significant progress in the treatment of burn injuries, the complex nature of burn trauma poses ongoing challenges in preventing and managing both systemic complications and complications specific to burn wounds [23,24]. In our study, including extensive burns with a >20% TBSA, we observed a high mortality rate; therefore, we traced a series of parameters well-known in the literature as negative prognosis factors in burn injuries, related both to the characteristics of the patient and the burn injury. The patients included in the present study had a poor prognosis based on their ABSI score (more than half of the patients had an ABSI score ≥ 10), their age (46.6% of the patients with age >60 years), their percentage of the total body surface area burned (with mean TBSA being 48.68 ± 20.08%), the presence of third degree-burns in 75% of the patients, and the presence of inhalation injuries in more than two-thirds of the patients.

Some of the survivors in our study showed a decrease in platelet count in the initial days after the burn injury, followed by an increase in platelet count to within the normal range during the next period, while the rest of the survivors showed normal platelet counts at any given time. On day 3, out of the 32 survivors, 14 patients (43.8% of survivors) had thrombocytopenia. On day 7, only six of them (28.8%) had thrombocytopenia. On day 14, all the survivors had normal platelet counts. Previous studies confirmed this rising trend in platelet count in survivors after the initial fall [3,9,10,25,26].

Compared with the survivors, the platelet count was significantly lower at any given time in the non-survivors group. In a study on 590 severe burn patients, Lin JC et al. also found that the platelet count was lower on day 1, day 3, and day 7 in non-survivors than in survivors [27].

Data from the literature showed that the platelet count decreased to the lowest level on day 3 after burn injury, the results which were also confirmed for the survivor group in our study [27,28]. The primary factor behind this alteration might be linked to heightened platelet activation and aggregation following a burn injury, leading to substantial platelet consumption. Additionally, fluid resuscitation may contribute to hemodilution in this context. In our study, non-survivors also showed a low platelet count on day 3 that persisted on days 7 and 14.

Regardless of the moment, patients with thrombocytopenia in our study died significantly more frequently. These findings are in concordance with the literature on burn patients and critically ill patients admitted to the ICU [10,26,29,30].

Previous studies on patients admitted to the adult intensive care units showed that late thrombocytopenia, meaning thrombocytopenia that persists for 14 days after initial intensive care unit admission, is often found to be a more reliable predictor of poor outcomes and death in critically ill patients compared to early thrombocytopenia, when platelet levels return to normal platelet counts by day 4. This suggests that the inability to recover or increase platelet counts in the later stages of critical illness may be associated with a worse prognosis [31,32]. In our study on burn patients, thrombocytopenia on day 3 increased the risk of death 2 times, that on day 7 increased the risk of death 5.8 times, that on day 14 increased the risk of death 6.1 times, that on day 21 increased the risk of death 9.6 times, and that on day 28 increased the risk of death 6.6 times.

The mean ABSI score was 10.16 ± 2.47 (range of 5–16). Patients with higher ABSI scores had thrombocytopenia on days 3, 7, and 14 (*p* < 0.001). There was no obvious difference in platelet levels in the following periods in relationship with the ABSI score.

In our study, patients with a higher TBSA burned (median = 50%, IQR = 35–70%) had thrombocytopenia on day 3 after the burn injury significantly more frequently compared to patients with a smaller TBSA burned (median = 35%, IQR = 27.5–40%). These results are in concordance with previous studies. Barbier et al.’s study, including 167 patients with severe burns, also showed that during the first 72 h, thrombocytopenia increased with the burned surface and had a nadir by day 4 [33].

Patients with a higher TBSA (median = 50%, IQR = 35–70%) also had thrombocytopenia on day 7 after the burn injury significantly more often than patients with a smaller TBSA burned (median = 35%, IQR = 25–42.5%). Analysis of data on day 14 and beyond revealed no statistically significant correlation between the TBSA and platelet count.

The mean overall age of the patient cohort in our study was 57.18 ± 18.33 (range of 20–90 years). Older patients had significantly lower platelet counts at any point in time, except on day 21, with a strong association in days 3 and 7 post-burn injury. Marck RE et al. [28] observed in a previous study on 244 patients that there is a difference in the course of the thrombocyte counts in three age categories (age < 18; age between 18 and 49; age > 49), meaning that at the lowest point, the youngest group exhibited notably elevated platelet counts, while at the highest point, the oldest group demonstrated a significantly reduced count. Their study also demonstrated that the oldest age group had significantly lower platelet values over a follow-up of 50 days compared to the other age categories.

Although a previous study conducted by Warner P. et al. on the pediatric population [30] showed that inhalation injury involvement was associated with clinically significant thrombocytopenia, our study could not confirm these results. In our study, patients with inhalation injury only had thrombocytopenia on day 7 post-burn more frequently than patients without inhalation injury.

During the dynamic evolution of burn injuries, systemic complications often occur, impacting the prognosis, with sepsis being the most severe. It is crucial to identify early signs of infection, especially since burn patients are immunocompromised [34]. We have also analyzed the dynamics of platelets with the occurrence of sepsis.

Previous studies suggested that monitoring the platelet count, along with other clinical and laboratory parameters, is an early warning sign of sepsis. Early recognition and prompt treatment of sepsis in burn patients are vital to improving their outcomes and reducing the risk of complications [35]. The proposed mechanisms of thrombocytopenia in sepsis consist of decreased production of platelets due to bone marrow failure, especially because of antibiotics and proton pump inhibitors [36,37], immune-associated thrombocytopenia due to the presence of platelet autoantibodies [38] or an unrestrained proliferation and activation of monocytes and macrophages that act as hemophagocytes [39], increased platelet sequestration in microvessels [39], disseminated intravascular coagulation with excessive consumption of platelets in sepsis [40], and hemodilution during massive crystalloid or colloid perfusion or blood product transfusions [41,42].

Correlating the clinical findings, paraclinical investigations, and confirmation by positive hemocultures, a diagnosis of sepsis was made in one-third of the patients in our study: in 18 of the deceased patients and 12 of the survivors. Thrombocytopenia was more frequent in patients with sepsis who did not survive, as compared to survivors, and did not normalize until the time of death. In our study group, we assessed the microorganisms involved in sepsis. A series of ESKAPE pathogens, including *Pseudomonas aeruginosa*, *Klebsiella pneumoniae*, *Acinetobacter baumannii*, and *Enterobacter* spp., were encountered predominantly in the non-survivor group of patients.

The Infectious Disease Society of America has identified six bacterial species as “ESKAPE pathogens.”: *Enterococcus faecalis*, Staphylococcus aureus, Klebsiella pneumoniae, Acinetobacter baumannii, Pseudomonas aeruginosa, and *Enterobacter* spp. [43] ESKAPE pathogens pose a therapeutic challenge due to their acquired antibiotic resistance. This resistance is becoming progressively more difficult to address, even with last-line antibiotics [44].

Reviewing the literature, several studies showed a prevalence of sepsis in burn patients varying from 26% [45] to 50.6% [46], 54% [47], or 65.5% [48]. This variation is influenced by factors such as the age and severity of burns within the study groups, as well as the definition used for sepsis [49]. Risk factors for sepsis were identified as follows: old age, TBSA > 20%, inhalation injury, male gender, and presence of comorbidities (cancer, immunosuppression, diabetes, liver disease, and kidney disease) [46,47,50]. While the overall mortality rate is consistently decreasing, the mortality of burn patients who develop sepsis remains around 34.4%, emphasizing the significance of addressing this issue to improve the overall outcomes of burn patients [45].

The early detection of septicemia in burn patients is paramount for preventing the devastating consequences of systemic inflammatory response syndrome and multiple organ dysfunction syndrome associated with sepsis. The decline in platelet count serves as a crucial early indicator, emphasizing the importance of vigilant monitoring. Future research efforts should focus on refining early detection parameters and interventions to improve the prognosis of burn patients at risk of septicemia.

Thrombocytopenia significantly influences clinical practice in major burn patients, particularly due to the frequent need for multiple surgical interventions (serial excision and grafting), which elevates the risk of increased bleeding. In addition, any element promoting bleeding, including thrombocytopenia, should be reduced to a minimum before burn surgery [23]. Unfortunately for surgical interventions, the literature reports that the platelet nadir level is on day 3 [28] or day 4 [33], which corresponds to the time when grafting procedures should start.

A limitation of this study is that we could not analyze thrombocytopenia in correlation with data regarding surgical intervention, transfusions, and the influence of medication on total platelet count due to the variability in the management of the patients, with differences based on the extent of the injuries and patient status, the need for multiple excision and skin-grafting procedures, and different operative moments. The lack of data uniformity prevented standardization.

## 5. Conclusions

Thrombocytopenia represents an early indicator of severe complications and outcome predictor in severely burned patients. It is correlated with recognized negative prognostic factors and also with sepsis occurrence. In the first days after the burn injury, patients died directly from the effects of burn shock or severe inhalation injury. Afterward, infections became the primary cause of death in patients who had survived the initial period. Regardless of the moment, patients with thrombocytopenia in our study died significantly more frequently. Compared with the survivors, the platelet count was significantly lower at any given time in the non-survivors group. Our study results confirm previous data from the literature, emphasizing the importance of platelet count in severe burn patients’ assessment and outcome prediction. Future research efforts should focus on refining early detection parameters and interventions to improve the prognosis of burn patients.

## Figures and Tables

**Table 1:** Characteristics of study patients.

Variables	Classification	Cases	Proportion (%)
Sex	Female	30	33.3%
	Male	60	66.7%
Age	≤40	23	25.5%
	41–60	25	27.8%
	61–80	31	34.4%
	>80	11	12.2%
Mechanism of injury	Flame	77	85.5%
	Scalds	8	8.9%
	Electrical burns	4	4.4%
	Contact burns	1	1.1%
Burn setting	Domestic accident	71	78.9%
	Work accident	3	3.3%
	Self-harm	12	13.3%
	Burning aggression	2	2.2%
	Undetermined	2	2.2%
TBSA	20–40	48	53.3%
	41–60	19	21.1%
	61–80	16	17.8%
	81–100%	7	7.8%
3rd-degree burns	No	22	24.4%
	Yes	68	75.6%
Inhalation injuries	No	26	28.9%
	Yes	64	71.1%

**Table 2:** The distribution of patients related to the presence of thrombocytopenia.

Thrombocytopenia—day 3/Survival (n = 88)	Survivors		Non-survivors		*p* *
	No.	Percent	No.	Percent	
Normal TPC	18	56.30%	11	19.60%	0.001
Thrombocytopenia	14	43.80%	45	80.40%	
Thrombocytopenia—day 7/Survival (n = 84)	Survivors		Non-survivors		*p* *
	No.	Percent	No.	Percent	
Normal TPC	26	81.30%	8	15.40%	<0.001
Thrombocytopenia	6	18.80%	44	84.60%	
Thrombocytopenia—day 14/Survival (n = 65)	Survivors		Non-survivors		*p* *
	No.	Percent	No.	Percent	
Normal TPC	31	100%	13	38.20%	<0.001
Thrombocytopenia	0	0%	21	61.80%	
Thrombocytopenia—day 21/Survival (n = 45)	Survivors		Non-survivors		*p* *
	No.	Percent	No.	Percent	
Normal TPC	24	96%	11	55%	0.002
Thrombocytopenia	1	4%	9	45%	
Thrombocytopenia—day 28/Survival (n = 36)	Survivors		Non-survivors		*p* *
	No.	Percent	No.	Percent	
Normal TPC	19	95%	10	62.50%	0.03
Thrombocytopenia	1	5%	6	37.50%	
Thrombocytopenia—day 45/Survival (n = 21)	Survivors		Non-survivors		*p* *
	No.	Percent	No.	Percent	
Normal TPC	10	90.90%	4	40%	0.024
Thrombocytopenia	1	9.10%	6	60%	
Thrombocytopenia—day 60/Survival (n = 13)	Survivors		Non-survivors		*p* *
	No.	Percent	No.	Percent	
Normal TPC	7	100%	2	33.30%	0.021
Thrombocytopenia	0	0%	4	66.70%	

n = number of patients left in the study group on a specific day after burn injury. * Fisher’s Exact Test.

**Table 3:** Survival of the patients related to the existence of thrombocytopenia.

Thrombocytopenia—day 3 post-burn Absent Present	Mean (95% CI)	Median (IQR)	*p* *
	65.1 (46.3–83.9)	63 (19–97)	0.025
	39.4 (28.9–49.9)	20 (10–60)	
Thrombocytopenia—day 7 post-burn Absent Present	Mean (95% CI)	Median (IQR)	*p* *
	80.1 (64.0–96.2)	97	<0.001
	30.1 (20.8–39.3)	16 (9–39)	
Thrombocytopenia—day 14 post-burn Absent Present	Mean (95% CI)	Median (IQR)	*p* *
	75.9 (62.9–88.9)	77	<0.001
	27.1 (17.2–37.1)	18 (16–26)	
Thrombocytopenia—day 21 post-burn Absent Present	Mean (95% CI)	Median (IQR)	*p* *
	83.9 (69.9–97.8)	97 (63–113)	<0.001
	34.3 (24.4–44.2)	27 (21–50)	
Thrombocytopenia—day 28 post-burn Absent Present	Mean (95% CI)	Median (IQR)	*p* *
	84.4 (69.8–99)	97 (63–113)	<0.001
	42.2 (30.8–53.7)	45 (27–60)	

* Log-rank test.

**Table 4:** The relationship between ABSI score and thrombocytopenia.

Platelet count—day 3 Normal TPC (*p* = 0.400 **) Thrombocytopenia (*p* = 0.125 **)	Mean ± SD	Median (IQR)	*p* * (*p* = 0.365 ***)
	8.41 ± 1.84	8 (7–9.5)	<0.001
	10.81 ± 2.15	11 (9–12)	
Platelet count—day 7 Normal TPC (*p* = 0.288 **) Thrombocytopenia (*p* = 0.320 **)	Mean ± SD	Median (IQR)	*p* * (*p* = 0.197 ***)
	8.32 ± 1.59	8 (7–9)	<0.001
	10.98 ± 2.05	11 (10–12)	
Platelet count—day 14 Normal TPC (*p* = 0.155 **) Thrombocytopenia (*p* = 0.13 7 **)	Mean ± SD	Median (IQR)	*p* * (*p* = 0.805 ***)
	8.80 ± 1.72	9 (8–10)	<0.001
	10.67 ± 1.68	11 (9.5–12)	

* Student *t*-Test, ** Shapiro–Wilk Test, *** Levene’s Test for Equality of Variances.

**Table 5:** Influence of age on platelet count levels.

Platelet count—day 3 post-burn	Mean ± SD	Median (IQR)	*p* *
Normal range (*p* = 0.134 **)	48.2 ± 16.7	44 (36–61.5)	0.002
Thrombocytopenia (*p* = 0.006 **)	61.1 ± 17.7	66 (44–76)	
* Mann–Whitney U Test, ** Shapiro–Wilk Test			
Platelet count—day 7 post-burn	Mean ± SD	Median (IQR)	*p* *
Normal range (*p* = 0.055 **)	48.7 ± 17.8	44 (34.7–61.2)	0.002
Thrombocytopenia (*p* = 0.039 **)	61.7 ± 16.8	65 (47.7–76.2)	
* Mann–Whitney U Test, ** Shapiro–Wilk Test			
Platelet count—day 14 post-burn	Mean ± SD	Median (IQR)	*p* * (*p* = 0.644 ***)
Normal range (*p* = 0.077 **)	52.4 ± 17.8	48.5 (38.5–69.5)	0.015
Thrombocytopenia (*p* = 0.389 **)	64 ± 16.9	64 (49–78.5)	
* Student *t*-Test, ** Shapiro–Wilk Test, *** Levene’s Test for Equality of Variances			
Platelet count—day 21 post-burn	Mean ± SD	Median (IQR)	*p* * (*p* = 0.039 ***)
Normal range (*p* = 0.090 **)	53.1 ± 19	47 (38–71)	0.056
Thrombocytopenia (*p* = 0.398 **)	63.2 ± 12.1	62.5 (49.7–73.5)	
* Welch *t*-Test, ** Shapiro–Wilk Test, *** Levene’s Test for Equality of Variances			
Platelet count—day 28 post-burn	Mean ± SD	Median (IQR)	*p* *
Normal range (*p* = 0.155 **)	54.1 ± 17.4	51 (40.5–71)	0.036
Thrombocytopenia (*p* = 0.013 **)	70.7 ± 18.8	76 (70–82)	
* Mann–Whitney U Test, ** Shapiro–Wilk Test			
Platelet count—day 45 post-burn	Mean ± SD	Median (IQR)	*p* * (*p* = 0.306 ***)
Normal range (*p* = 0.345 **)	52.2 ± 18.1	49 (38.7–72.2)	0.019
Thrombocytopenia (*p* = 0.503 **)	72.1 ± 13.4	76 (59–82)	
* Student *t*-Test, ** Shapiro–Wilk Test, *** Levene’s Test for Equality of Variances			
Platelet count—day 60 post-burn	Mean ± SD	Median (IQR)	*p* *
Normal range (*p* = 0.073 **)	51 ± 18.2	44 (37.5–73.5)	0.020
Thrombocytopenia (*p* = 0.003 **)	74 ± 15.3	81.5 (58.5–82)	
* Mann–Whitney U Test, ** Shapiro–Wilk Test			

**Table 6:** Comparison of total burn surface area burned relative to the existence of thrombocytopenia.

Thrombocytopenia—day 3 post-burn	Mean TBSA ± SD	Median TBSA (IQR)	*p* *
Absent (*p* < 0.001 **)	39.3 ± 15.4	35 (27.5–40)	0.004
Present (*p* = 0.006 **)	51.9 ± 19.7	50 (35–70)	
Thrombocytopenia—day 7 post-burn	Mean TBSA ± SD	Median TBSA (IQR)	*p* *
Absent (*p* = 0.004 **)	38.3 ± 13.1	35 (25–42.5)	0.001
Present (*p* = 0.011 **)	52.6 ± 20	50 (35–70)	

* Mann–Whitney U test, ** Shapiro–Wilk test.

**Table 7:** Distribution of patients related to post-burn thrombocytopenia and the existence of third-degree burns and inhalation injury.

TPC—day 3	Without 3rd-degree burns	3rd-degree burns	*p* *	No inhalation injury	Inhalation injury	*p* *
Normal TPC	12 patients (54.5%)	17 patients (25.8%)	0.019	12 patients (46.2%)	17 patients (27.4%)	0.135
Thrombocytopenia	10 patients (45.5%)	49 patients (74.2%)		14 patients (53.8%)	45 patients (72.6%)	
TPC—day 7	Without 3rd-degree burns	3rd-degree burns	*p* *	No inhalation injury	Inhalation injury	*p* *
Normal TPC	14 patients (63.6%)	20 patients (32.3%)	0.013	15 patients (60%)	19 patients (32.2%)	0.028
Thrombocytopenia	8 patients (36.4%)	42 patients (67.7%)		10 patients (40%)	40 patients (67.8%)	

* Fisher’s Exact Test.

## Data Availability

The dataset is available on request from the authors.
